# A novel necroptosis-related lncRNAs signature for survival prediction in clear cell renal cell carcinoma

**DOI:** 10.1097/MD.0000000000030621

**Published:** 2022-09-30

**Authors:** Liwen Zhao, Huaijing Luo, Xingmo Dong, Zhihui Zeng, Jianlong Zhang, Yi Yi, Chaolu Lin

**Affiliations:** a Department of Urology, Longyan First Affiliated Hospital of Fujian Medical University, Longyan City, China.

**Keywords:** clear cell renal cell carcinoma, immune microenvironment, lncRNA, necroptosis, prognosis

## Abstract

Clear cell renal cell carcinoma (ccRCC) is the most common kind of kidney cancer with poor prognosis. Necroptosis is a newly observed type of programmed cell death in recent years. However, the effects of necroptosis-related lncRNAs (NRlncRNAs) on ccRCC have not been widely explored. The transcription profile and clinical information were obtained from The Cancer Genome Atlas. Necroptosis-related lncRNAs were identified by utilizing a co-expression network of necroptosis-related genes and lncRNAs. Univariate Cox regression, least absolute shrinkage, and selection operator regression and multivariate Cox regression were performed to screen out ideal prognostic necroptosis-related lncRNAss and develop a multi-lncRNA signature. Finally, 6 necroptosis-related lncRNA markers were established. Patients were separated into high- and low-risk groups based on the performance value of the median risk score. Kaplan–Meier analysis identified that high-risk patients had poorer prognosis than low-risk patients. Furthermore, the area under time-dependent receiver operating characteristic curve reached 0.743 at 1 year, 0.719 at 3 years, and 0.742 at 5 years, which indicating that they can be used to predict ccRCC prognosis. In addition, the proposed signature was related to immunocyte infiltration. A nomogram model was also established to provide a more beneficial prognostic indicator for the clinic. Altogether, in the present study, the 6-lncRNA prognostic risk signature are trustworthy and effective indicators for predicting the prognosis of ccRCC.

## 1. Introduction

Renal cell carcinoma (RCC) is the second most common urological tumor. In American, about 76,080 new cases of kidney cancer will be diagnosed in 2021, and about 13,780 people will die from this disease.^[1]^ The morbidity and mortality of RCC has increased during the past several years.^[2]^ Furthermore, the 5-year survival rate is 97%, 87%, 69%, and 14% for patients in stages I, II, III, and IV, respectively.^[3]^ Unfortunately, approximately 30% patients experience metastatic lesions at the time of diagnosis.^[4]^ In addition, no evidences identified that adjuvant therapies such as chemotherapy, vaccines, or cytokines are effective after surgery.^[5]^ The natural clinical course varies in RCC, which has led to the development of different prognostic models for the assessment of the patient’s individual risk.^[6]^ Therefore, there is a growing need to develop new prognostic and predictive biomarkers to identify potentially high-risk RCC patients.

Necroptosis, a newly observed programmed cell death, which is characterized by necrotic cell death morphology and activation of autophagy.^[7]^ Necroptosis has emerged as a crucial pathologic process involved in many diseases, including neurologic, cardiovascular, pulmonary, and gastrointestinal systems.^[8]^ It is now known that receptor interacting protein kinase 1 and 3 (RIPK1 and RIPK3) and the mixed lineage kinase domain-like constitute the core of the necroptosis machinery.^[9]^ Necroptotic cells were shown to initiate adaptive immunity by providing both antigens and inflammatory stimuli for dendritic cells (DCs), which in turn activate CD8+ T cells and antitumor immunity.^[10]^

Long noncoding RNAs (lncRNAs) consist with at least 200 nucleotides in length.^[11]^ LncRNAs regulate gene expression and pathophysiological processes at the epigenetic, transcriptional, and post-transcriptional levels generally via gene imprinting, histone modification, chromatin remodeling, transcriptional interference, alternative splicing, and cell cycle control.^[12]^ LncRNAs not only play important roles in biological regulatory mechanisms, but make great functions in multiple diseases, including liver diseases, cancers, and cardiovascular diseases.^[13–16]^ There are currently many studies identified that lncRNAs participated in multiple cancer processes, such as proliferation, invasion, metastasis, and the responses to therapies.^[17]^ At the same time, lncRNAs can also play an important role in mediating necroptosis. Linc00176 in hepatocellular carcinoma regulates cell cycle and induced necroptosis by releasing tumor suppressor microRNAs, such as microRNA(miR)-9 and microRNA-185.^[18]^ Upregulated Tp53-regulated inhibitor of necrosis under glucose starvation protects cancer cells from necroptosis under glucose starvation via inhibiting STRAP-GSK3β-NF-κB axis.^[19,20]^ However, it remained unclear whether necroptosis-related lncRNAs were associated with the prognosis of renal cell carcinoma patients.

## 2. Materials and Methods

### 2.1. Data collection

The RNA transcriptome datasets (HTSeq-Counts and HTSeq-FPKM) and the relevant clinical information were extracted using The Cancer Genome Atlas-Kidney Renal Clear Cell Carcinoma (TCGA-KIRC) database from 611 individuals (72 normal samples and 539 tumor samples). Table S1, Supplemental Digital Content 1, http://links.lww.com/MD/H332, which illustrates clinical features of the patients, shows the clinical features of the patients. Then, we converted the FPKM value to the transcripts perkilobase million value of the synthetic matrix by data.table, tibble, dplyr, and tidyr R packages. As a result, we got 2 synthetic data matrices. The Counts value matrix was just for identifying differentially expressed lncRNAs, while the transcripts perkilobase million value matrix was for the other analyses. To reduce statistical bias in this analysis, clear cell renal cell carcinoma patients with short overall survival (OS) values (<30 days) were excluded. Consequently, we extracted 512 patients who had sufficient gene expression profiling along with OS data from the TCGA dataset for subsequent analysis. The necroptosis gene set M24779.gmt and M25944.gmt were downloaded from the Gene Set Enrichment Analysis (GSEA) (http://www.gsea-msigdb.org/gsea/index.jsp). In addition, with previous reports about necroptosis, we finally obtained the profile of 68 necroptosis-related genes (see Table S2, Supplemental Digital Content 2, http://links.lww.com/MD/H333, which illustrates the necroptosis-related genes we get).

### 2.2. Selection of necroptosis-related lncRNAs

The “limma” package in R software was used to identify the differentially expressed genes and lncRNAs (DElncRNAs) between clear cell renal cell carcinoma (ccRCC) and normal tissues. Then, the gene was excluded if the sum of gene expression level for each sample is <1 or it is an unrecognized gene. *P* value <.05, false discovery rate <0.05, and |log_2_(fold change, FC) ≥1 were considered significantly different, including both upregulated and downregulated. Twenty-one of the 68 necroptosis-related genes were differentially expressed (see Table S3, Supplemental Digital Content 3, http://links.lww.com/MD/H334, which illustrates the differentially expressed necroptosis-related genes). Then, the correlation analysis was performed between DElncRNAs and the 21 necroptosis-related genes. We selected necroptosis-related lncRNAs (NRlncRNAs) using the thresholds of *P* < .01 and correlation coefficient *R* > 0.5. The Kyoto Encyclopedia of Genes and Genomes pathway analysis was performed to identify the signaling pathways associated with the 21 necroptosis-related genes and *P* value of <.05 and false discovery rate of <0.25 were considered statistically significant.

### 2.3. The development of a predictive signature for necroptosis-related lncRNAs

First, potential prognostic lncRNAs were identified by univariate Cox regression using the threshold of *P* < .05. Subsequently, overfitting genes were reduced by least absolute shrinkage and selection operator (LASSO) regression. Finally, we established a prognostic model by multivariate Cox regression prognostic outcomes of ccRCC. The risk score for ccRCC cases was calculated as follows: risk score = (NRlncRNA 1 expression × coefficient) + (NRlncRNA 2 expression × coefficient) +… + (NRlncRNA n expression × coefficient). Meanwhile, the cases were classified into low- or high-risk groups based on the median value. Moreover, we utilized the R package “rms” to construct a nomogram that integrated the risk score of the signature and clinical factors (age, gender, and clinical stage). R packages “survival” and “survminer” were introduced to evaluate OS based on Kaplan–Meier method. R package “survivalROC” was applied for the generation of receiver operating characteristic (ROC) curve, while the area under the ROC curves (AUCs) of risk score, grade, and stage were used to evaluate the accuracy for predicting OS. Principal component analysis was introduced for the exploration of group distribution using R package “scatterplot3d.”

### 2.4. Infiltrating immune cell analysis of the prognostic signature

In order to conduct immune infiltration analysis, we calculate the immune infiltration statuses among the ccRCC patients including TIMER, CIBERSORT, CIBERSORT-ABS XCELL, QUANTISEQ, MCP-COUNTER, and EPIC on TIMER2.0 (http://timer.cistrome.org/). CIBERSORT and ESTIMATE were used to estimate immune score and stromal score. Using a heatmap, the disparities in the immunological response were discovered. Furthermore, single sample gene set enrichment analysis (ssGSEA) was conducted to assess immune cell subpopulations between the 2 groups as well as measuring their capacity to defend tumor infiltration. Potential immune checkpoint has been found in the literature previously.

### 2.5. Analysis of the risk model performance in clinical chemotherapy

To assess the signature in the clinical utility of ccRCC treatment, 8 chemotherapeutic and targeted drugs in ccRCC treatment were selected, including axitinib, bortezomib, cisplatin, gefitinib, sorafenib, sunitinib, temsirolimus, and vinblastine. We analyzed the half inhibitory concentration (IC50) of chemotherapeutic and targeted drugs using “pRRophetic” R package.

## 3. Results

### 3.1. Identification of differentially expressed NRlncRNAs

**Figure 1.**
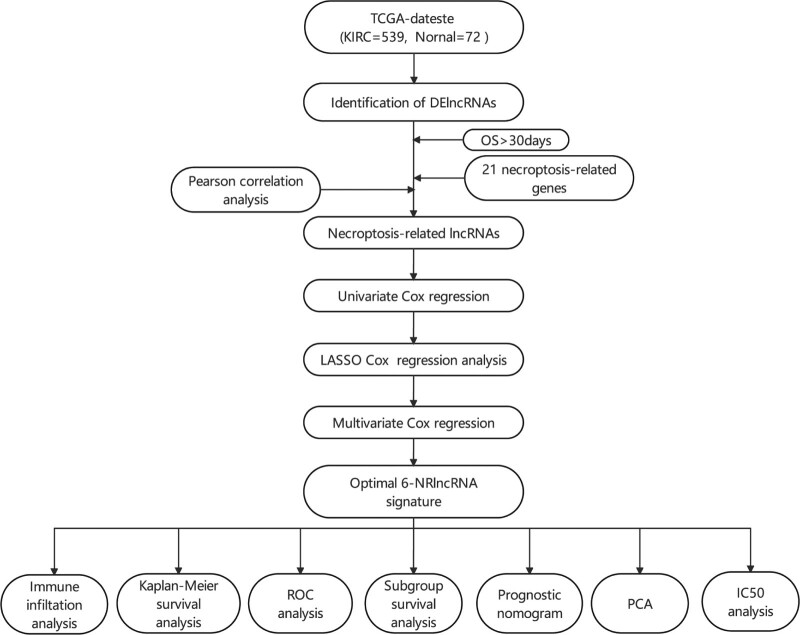
Flowchart of the study. DElncRNAs = differentially expressed long noncoding RNAs, IC50 = half inhibitory concentration, LASSO = least absolute shrinkage, and selection operator, NRlncRNA = necroptosis-related long noncoding RNA, OS = overall survival, PCA = principal component analysis, TCGA = The Cancer Genome Atlas.

**Figure 2.**
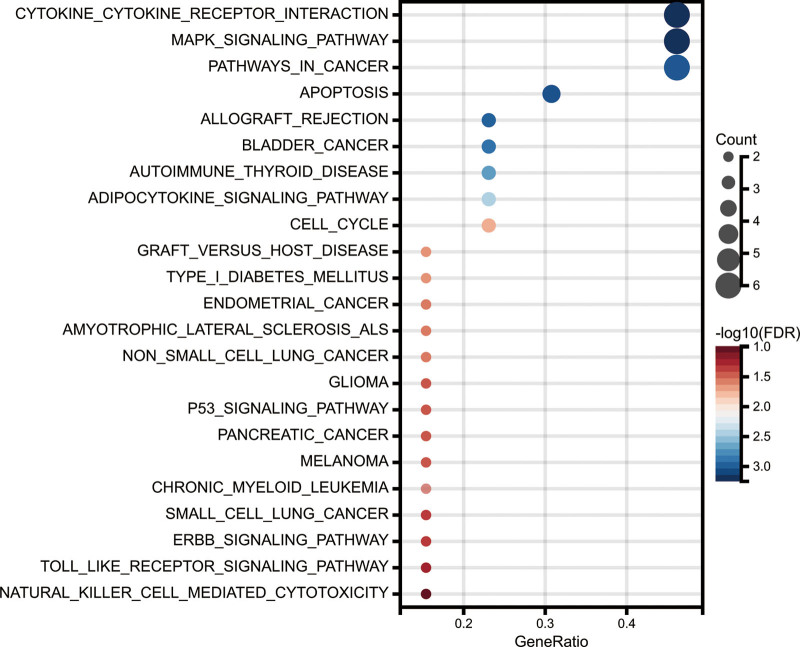
KEGG enrichment analyses of necroptosis-related lncRNAs. KEGG = kyoto encyclopedia of genes and genomes, lncRNAs = long noncoding RNAs.

The flowchart of the study is presented in Figure 1. Throughout the TCGA-KIRC data, 2916 lncRNAs were identified. Using cutoff values of |log_2_FC| > 1 and *P* < .05, 1271 DElncRNAs were identified between 539 KIRC and 72 noncancerous samples. According to the expression of 21 necroptosis-related genes and DElncRNAs between normal and tumor samples, we finally got 140 necroptosis-related lncRNAs (*P* < .01 and Pearson correlation coefficient |*R*| > 0.5; see Table S4, Supplemental Digital Content 4, http://links.lww.com/MD/H335, which illustrates the necroptosis-related lncRNAs). In addition, the necroptosis-related genes were mostly involved in the cytokine–cytokine receptor interaction and MAPK signaling pathway, according to Kyoto Encyclopedia of Genes and Genome analysis (Fig. 2).

### 3.2. Construction of the model

**Figure 3.**
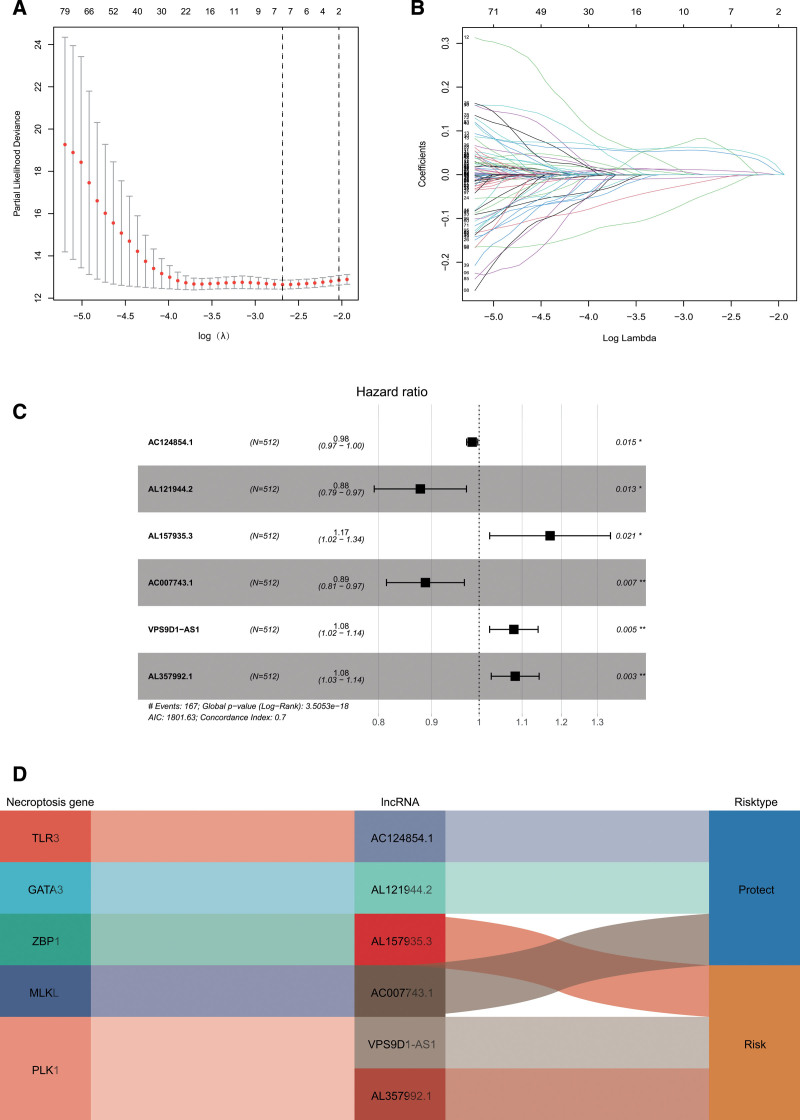
Construction of risk signature by LASSO and Cox regression analysis. (A) Cross-validation in the LASSO regression. (B) LASSO regression of the OS-related genes. (C) Multivariate Cox regression analysis revealed that the forest plot of necroptosis-associated lncRNAs is substantially associated with OS in ccRCC patients. (D) The Sankey diagram of necroptosis genes and related lncRNAs. ccRCC = clear cell renal cell carcinoma, LASSO = least absolute shrinkage, and selection operator, lncRNAs = long noncoding RNAs, OS = overall survival.

**Figure 4.**
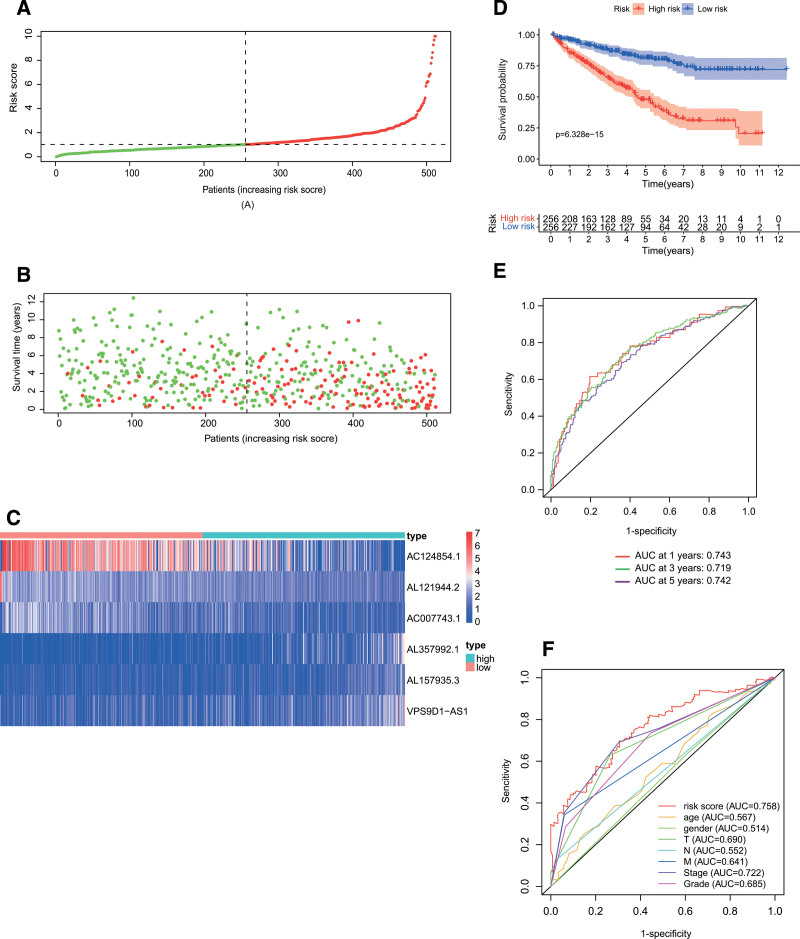
Prognosis value of the 6 necroptosis-related lncRNAs model. (A) Exhibition of necroptosis-related lncRNAs model based on risk score. (B) Survival time and survival status between low- and high-risk groups. (C) The heat map of 6 lncRNAs expression. (D) K–M curves for the OS of patients in 2 groups. (E) AUC for predicting the survival rate of ccRCC patients after 1, 3, and 5 years. (F) ROC curves revealed the predictive efficiency of the risk score. AUC = area under time-dependent ROC curve, ccRCC = clear cell renal cell carcinoma, lncRNAs = long noncoding RNAs, OS = overall survival, ROC = receiver operating characteristic.

According to univariate Cox regression analysis, we found 53 necroptosis-related lncRNAs significantly correlated with OS (*P* < .05; see Table S5, Supplemental Digital Content 5, http://links.lww.com/MD/H336 which illustrates the OS-related necroptosis-related lncRNAs). To avoid overfitting the prognostic signature, we performed the LASSO regression on these lncRNAs and extracted 7 lncRNAs related to necroptosis in ccRCC. In the multivariate analysis, 6 lncRNAs (AC124854.1, AL121944.2, AL157935.3, AC007743.1, VPS9D1-AS1, and AL357992.1) were found to be independent prognostic predictors of ccRCC (Fig. 3). These 6 lncRNAs were utilized as signature lncRNAs related to necroptosis. The formula of the risk score was as follows: Risk score = (−0.0154 × AC124854.1) + (−0.1303 × AL121944.2) + (0.1574 × AL157935.3) + (−0.1191 × AC007743.1) + (0.0771 × VPS9D1-AS1) + (0.08 × AL357992.1). With the risk score formula, the distribution of risk score, the survival status, survival time, and the relevant expression standards of these lncRNAs of patients were compared between low- and high-risk groups of ccRCC patients. These all indicated the high-risk group had worse prognoses (Fig. 4A–D). As shown in Figure 4E, the AUC was 0.743 at 1 year, 0.719 at 3 years, and 0.742 at 5 years. Furthermore, the lncRNA signature had an AUC value of 0.758, outperforming conventional clinicopathological characteristics in predicting ccRCC prognosis (Fig. 4F).

### 3.3. Subgroup analysis of the NRlncRNA prognostic model

**Figure 5.**
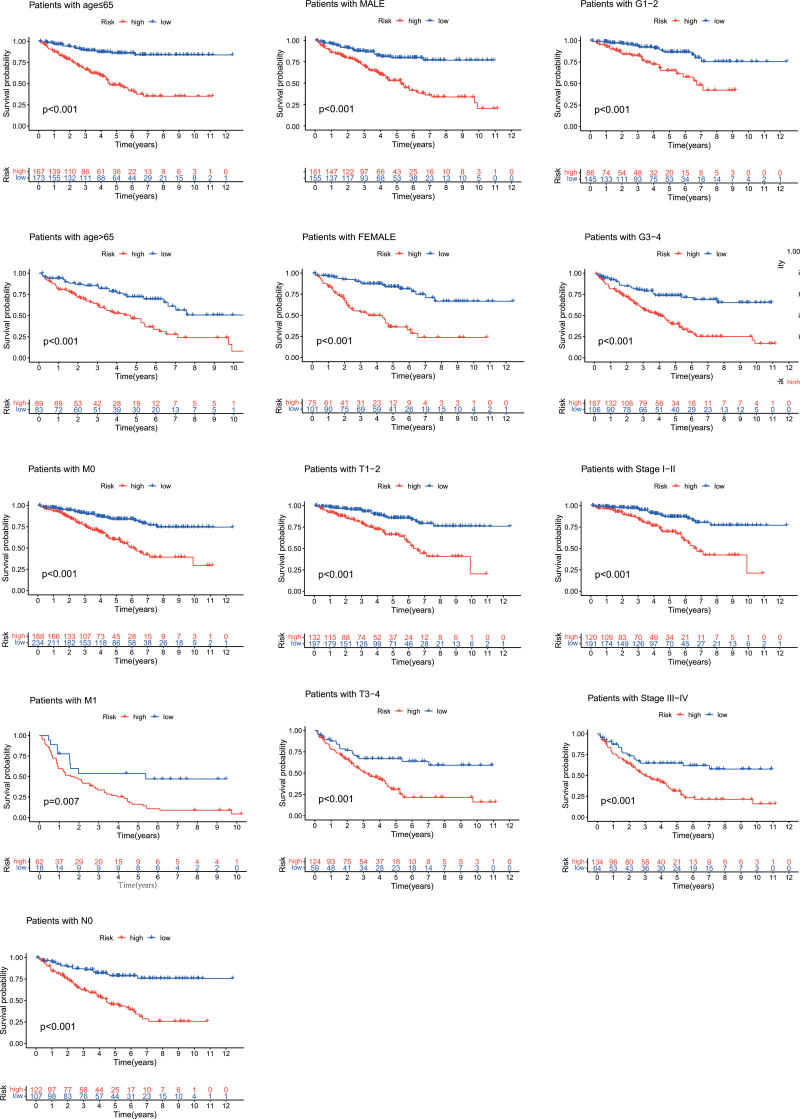
Kaplan–Meier survival curves of OS prognostic value stratified by age, gender, grade, stage, T, N, or M between low- and high-risk groups. OS = overall survival.

We further performed subgroup survival analysis to determine whether the prognostic model could predict OS for patients based different clinical features. These subgroups were separated by age (≤65 or >65), gender (male or female), tumor grade (grade 1–2 or grade 3–4), clinical stage (stage I–II or stage III–IV), tumor (T, T1–2, or T3–4), node (N), and metastasis (M, M0, or M1). As shown in Figure 5, high-risk patients exhibited inferior OS rates compared to low-risk patients according to age, gender, grade, and clinical stage.

### 3.4. Construction and validation of a prognostic nomogram

**Figure 6.**
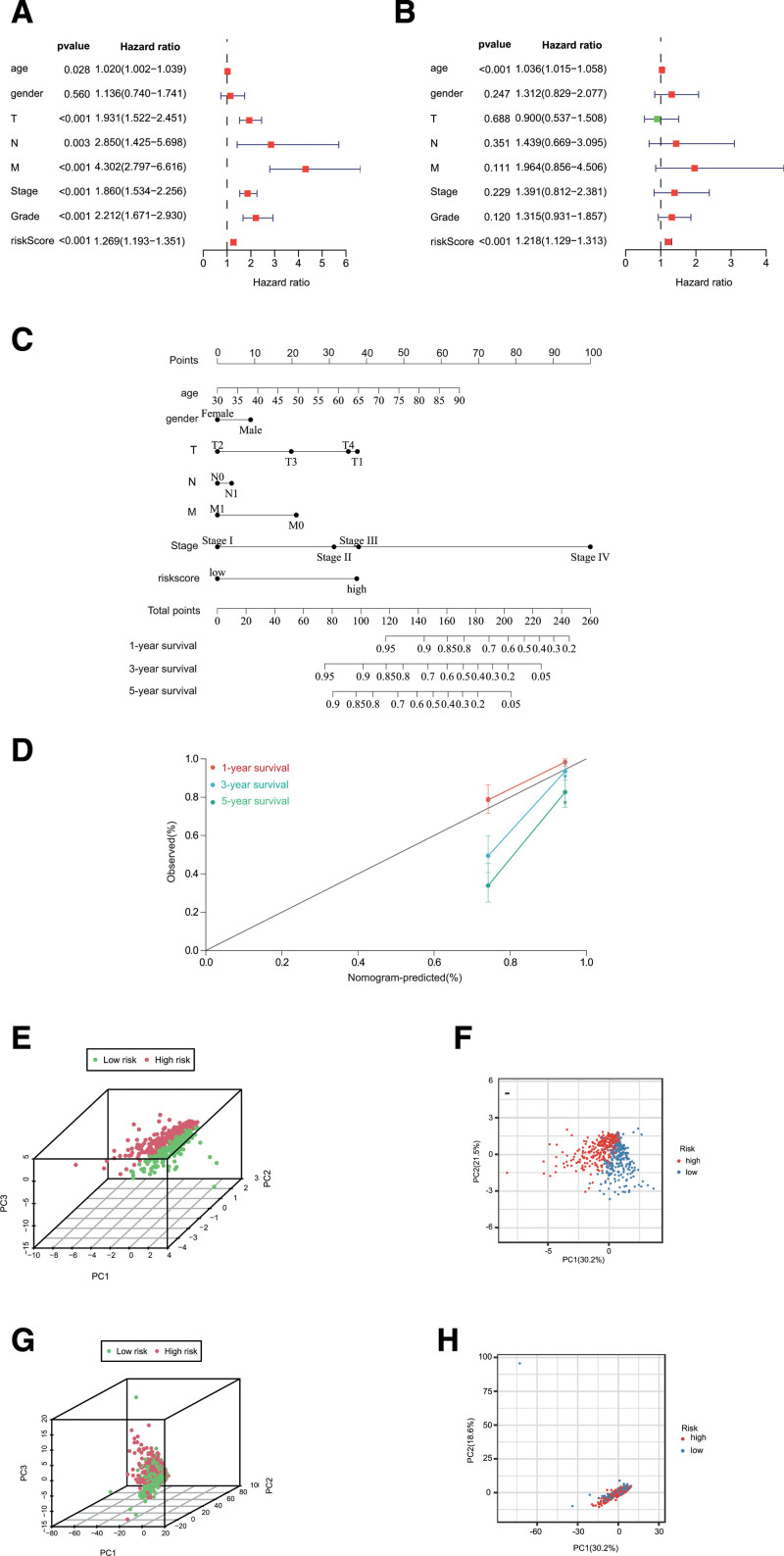
Nomogram and assessment of the risk model. (A) Univariate and (B) multivariate Cox regression methods for independent prognostic analysis of risk model; (C) nomogram constructed to predict OS rates at 1, 3, and 5 y; (D) the nomogram calibration curves on consistency between predicted and observed 1-, 3-, and 5-y survival. (E–H) PCA of risk groups based on the necroptosis-associated gene sets. OS = overall survival, PCA = principal components analysis.

To verify that our constructed prognostic signature could independently predict the prognosis of ccRCC cases, univariate and multivariate Cox regression analyses were performed. As revealed by univariate analysis, clinical stage (*P* < .001), T stage (*P* < .001), risk score (*P* < .001), N stage (*P* = .003), and M stage (*P* < .001) predicted dismal OS. Moreover, our multivariate Cox regression results validated the independence of our constructed prognostic model for predicting ccRCC prognosis (Fig. 6A, B). Next, we combined the risk score and other clinicopathologic parameters to develop a novel nomogram to predict OS rates for ccRCC cases at 1, 3, and 5 years (Fig. 6C, D). Besides, principal component analysis showed that the high-risk and low-risk samples clustered separately in 3- and 2-dimensional space based on the 6-NRlncRNA expression (Fig. 6E, F). However, there was no observable separation between high-risk and low-risk samples on the basis of the whole necroptosis-related lncRNA expression profiles (Fig. 6G, H). The result demonstrated a distinguishing distribution pattern of the high-risk and low-risk groups grounded on the necroptosis-related signature, reflecting that our constructed signature had more discriminatory ability to identify the difference in necroptosis phenotype among the samples when compared to the whole necroptosis-related lncRNA expression profiles.

### 3.5. Immune infiltration analysis

**Figure 7.**
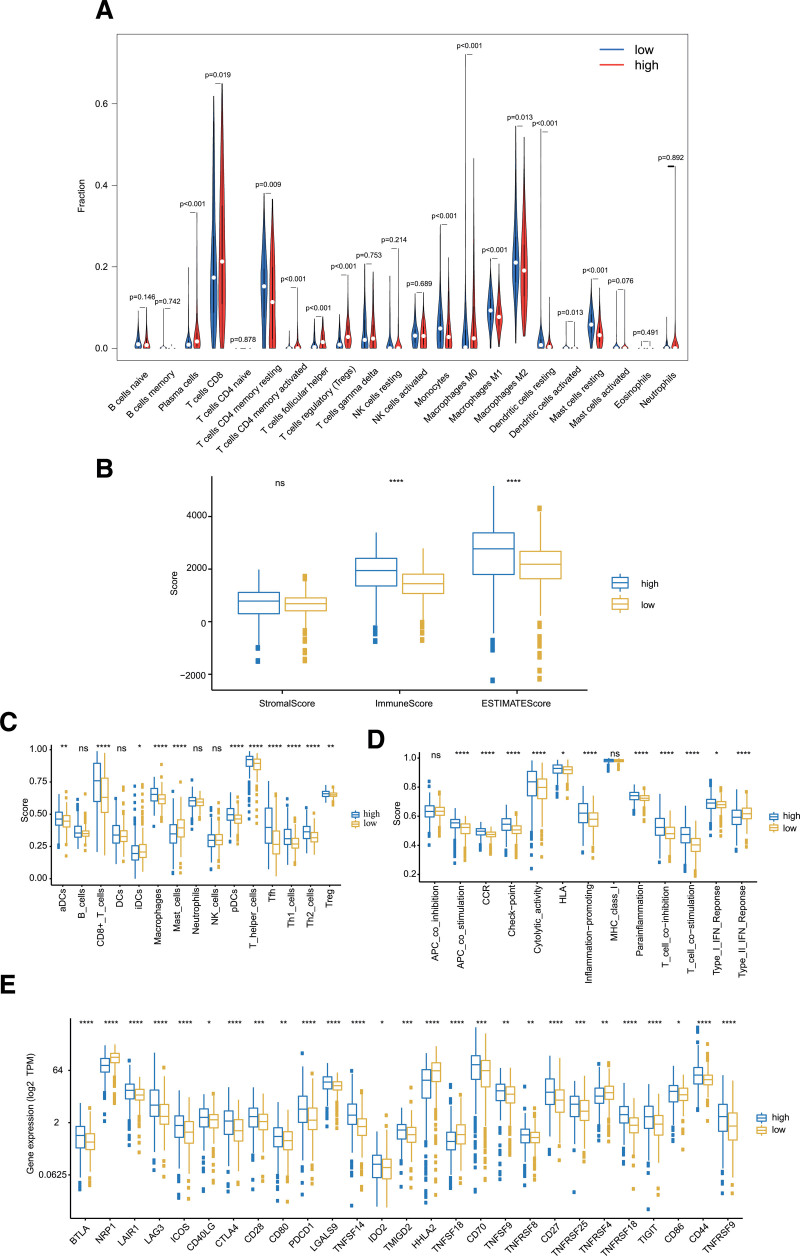
Immune cells infiltration analysis. (A) The vioplots showed that 22 immune cells content in the high- and low-risk groups. (B) ESTIMATE comparison of stromal, immune, and tumor purity scores in high- and low-risk groups. (C, D) The ssGSEA scores of immune cells and immune functions in high- and low-risk groups. (E) Expression of immune checkpoints in high-risk and low-risk groups(*P < .05; **P < .01; ***P < .001; ****P < .0001). ssGSEA = single sample gene set enrichment analysis.

**Figure 8.**
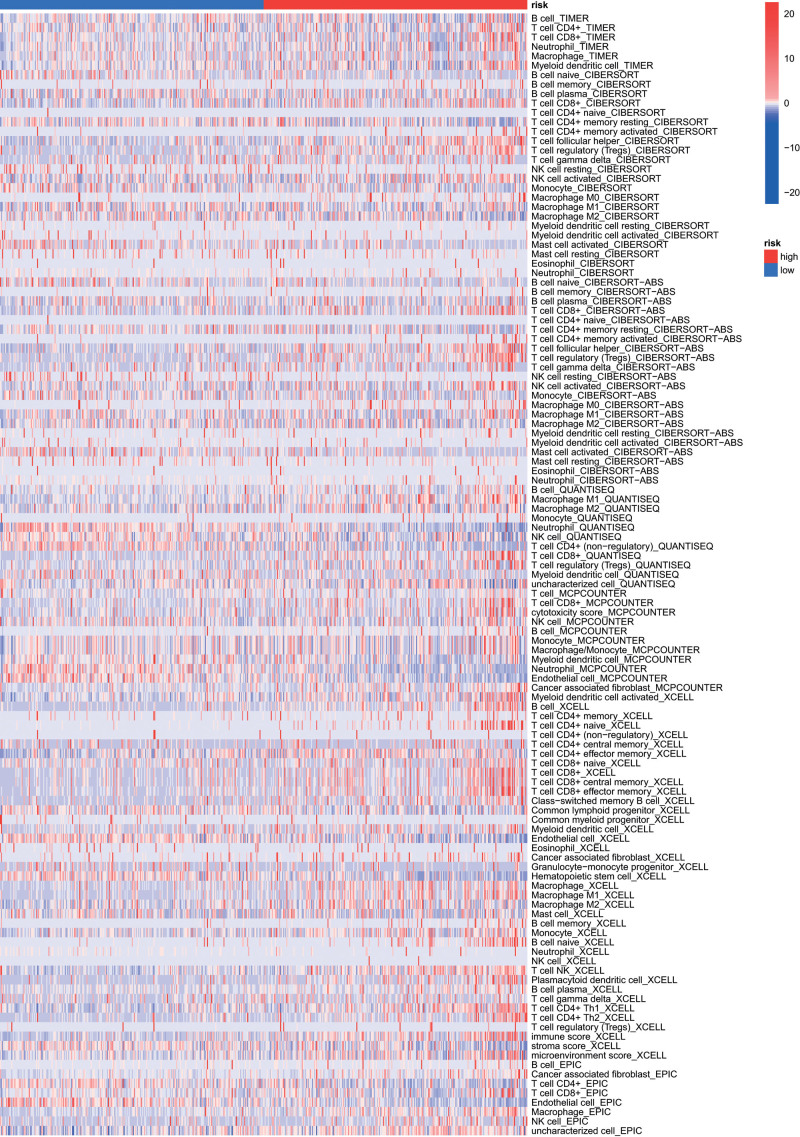
The heatmap of immune responses based on different algorithms among the high- and low-risk groups.

We evaluated the infiltration of the 22 types of immune cells in the TCGA database by the CIBERSORT algorithm estimation and found that 13 types of immune cells were significantly different between the high- and low-risk groups (*P* < .05; Fig. 7A). Figure 7B shows that the immune score was significantly higher in the high-risk group (*P* < .001), whereas the stromal score was not statistically significant in the high- and low-risk groups. Moreover, the combined ESTIMATE score was higher in the high-risk group than in the low-risk group (*P* < .05). Concerning the comparison of the single sample GSEA scores for immune cells and immune functions, 9 immune cells, such as type 1 T helper cells (Th1_cells), and most immune functions, such as cytolytic_activity, had more relations with high-risk patients (Fig. 7C, D). In addition, the heatmap of immune responses based on different algorithms is illustrated in Figure 8. The results demonstrated that most immune cells expressed at a higher level in the high-risk group than in the low-risk group. Furthermore, most immune checkpoints expressed more activity in high-risk patients, such as CTLA4, LAG3, and PDCD1 (Fig. 7E).

### 3.6. Correlation between the risk model and drug sensitivity

**Figure 9.**
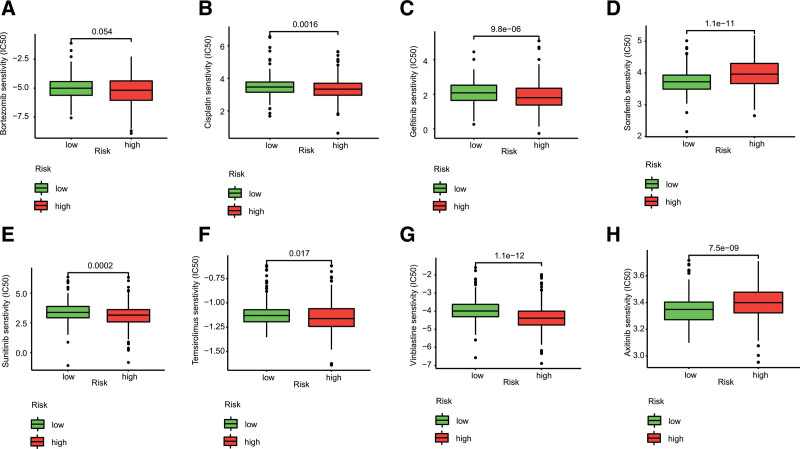
Lower IC50 of chemotherapeutic drugs between the high- and low-risk group based on the necroptosis-related lncRNA signature. (A) Bortezomib, (B) cisplatin, (C) gefitinib, (D) sorafenib, (E) sunitinib, (F) temsirolimus, (G) vinblastine, (H) axitinib. IC50 = half inhibitory concentration, lncRNAs = long noncoding RNAs.

The responsive predictive values of risk model for chemotherapy and targeted drugs were calculated by IC50 values (Fig. 9). Compared with the low-risk group, the IC50 value of cisplatin, gefitinib, sunitinib, temsirolimus, and vinblastine was significantly lower in the high-risk group, which means patients with higher risk score were more sensitive to these drugs.

## 4. Discussion

Necroptosis, a form of programmed cell death, has so far hardly been focused on with regard to a future treatment of cancer patients and may emerge as a novel and effective approach to eliminate tumor cells.^[21]^ However, the role of necroptosis in cancer is complicated. It is reported that necroptosis can elicit adaptive immune responses that may defend against tumor progression; however, the recruited inflammatory response may also promote tumorigenesis and cancer metastasis.^[22]^ The previous study identified that most high-grade ccRCC cells express increased amounts of RIPK1 and RIPK3 and are poised to undergo necroptosis in response to TNF receptor 1 (TNFR1) signaling.^[23]^ Furthermore, necroptosis is correlated with microvascular invasion which has potential prognostic value in RCC.^[24]^ In addition, necroptosis is identified to be promising novel target for cancer therapies.^[10]^ Accumulating evidence has shown that aberrant expression of lncRNAs would affect the prognosis of cancer patients. Therefore, it is meaningful to screen ideal necroptosis-related lncRNAs as biomarkers and construct a prognostic model to predict the prognosis of ccRCC patients.

In this study, novel prognostic necroptosis-related lncRNAs were identified through differentially expressed analysis, univariate Cox regression, LASSO regression, and multivariate Cox regression. Finally, 6 ideal novel risky NRlncRNAs (AC124854.1, AL121944.2, AL157935.3, AC007743.1, VPS9D1-AS1, and AL357992.1) were shown to be independent prognostic factors for ccRCC. Fa et al^[25]^ demonstrated that VPS9D1-AS1 could up-regulate SEC61A1 through sponging miR-491-5p and facilitate cell proliferation, migration and stemness in hepatocellular carcinoma cells. Also, a recent study revealed that VPS9D1-AS1 promoted the oncogenicity of colorectal cancer cells by acting as a molecular sponge of miR-525-5p and increasing the expression of HMGA1.^[26]^ In lung adenocarcinoma, VPS9D1-AS1 was reported to promote malignant progression by targeting miR-30a-5p/KIF11 axis.^[27]^ AC124854.1 was identified to serve as a prognostic and diagnostic biomarkers for ccRCC.^[28]^ For AL121944.2, AL157935.3, AC007743.1, and AL357992.1, the role of lncRNAs in cancer has not been reported so far.

Immunotherapy is the new backbone in the therapeutic landscape of renal cell carcinoma.^[29]^ Immune cell infiltration is an important prerequisite for the effectiveness of immunotherapy.^[30]^ In this study, the high-risk group had a significantly elevated immune score and ESTIMATE score. Zeng et al^[31]^ found that high immune score was associate with poor prognosis in gastric cancer. To investigate the infiltration of immune cells, we compared the contents of immune cell content groups with different risk scores and found that plasma cells, T cells CD8, T cells CD4 memory activated, T cells follicular helper, T cells regulatory (Tregs), and macrophages M0 were significantly higher than those in the low-risk group, while T cells CD4 memory resting, monocytes, macrophages M1, macrophages M2, DCs resting, DCs activated and mast cells resting were higher in the low-risk group than in the high-risk group. A high immune infiltration level of T cells CD8, T cells follicular helper, and Tregs was associated with poorer prognosis of ccRCC.^[32]^ Tregs play a key role for the maintenance of immune homeostasis and peripheral tolerance.^[33]^ Increased Tregs in the tumor microenvironment is associated with higher grade and stage in renal cell carcinoma.^[30]^ In addition, tracking the role of Tregs in peripheral blood in patients with renal cell carcinoma is helpful in understanding the immune response of the antitumor and predicting the impact of immunotherapy.^[34]^ It is generally believed that macrophages M1 and macrophages M2 play tumor antagonizing and tumor promoting roles, respectively, in tumor immunotherapy.^[33]^ However, we found that macrophages M2 with high components of the tumor microenvironment in ccRCC indicated better OS. This contradiction needs further study to be explained.

To better assess the clinical feasibility of the risk model, we analyzed the efficacy of the presented model in chemotherapy and targeted drugs. The results indicated that high-risk patients can get more benefits from chemotherapy and targeted drugs except sorafenib and axitinib. Therefore, we believe that the risk model we established can help identify better treatment strategies for individual patients with advanced ccRCC.

In this study, we have identified a novel necroptosis-related lncRNAs which could be biomarkers for ccRCC. Limitation of our study should be acknowledged. The original dataset for setting up the lncRNA-related model was merely retrieved from the TCGA database. So, the results needed to be validated in clinical samples.

## 5. Conclusion

The present study developed a 6-NRlncRNA signature that offers valuable clinical application for prognostic forecasting.

## Acknowledgments

This study was supported by Startup Fund for scientific research, Fujian Medical University (grant number: 2020QH1332). The information of this study here is obtained by The Cancer Genome Atlas (TCGA) database. We are grateful to them for the source of data used in our study.

## Author contributions

Conceptualization: Liwen Zhao, Chaolu Lin.

Data curation: Liwen Zhao, Huaijing Luo.

Formal analysis: Liwen Zhao, Xingmo Dong.

Investigation: Liwen Zhao, Zhihui Zeng, Jianlong Zhang.

Methodology: Liwen Zhao, Yi Yi.

Project administration: Chaolu Lin.

Software: Liwen Zhao, Yi Yi.

Writing - original draft: Liwen Zhao.

Writing - review and editing: Liwen Zhao, Yi Yi.

## Supplementary Material


