# Supplementary material for: A novel necroptosis-related lncRNAs signature for survival prediction in clear cell renal cell carcinoma
Source: Medicine (Baltimore). 2022 Sep 30;101(39):e30621. doi: 10.1097/MD.0000000000030621 (PMC9524942; doi:10.1097/MD.0000000000030621)
Supplement: Supplementary file 1 [file medi-101-e30621-s001.pdf]

Table S1. Patients' clinical features from the TCGA dataset.

Variable	Number of samples
**Gender**	
Male	346
Female	191
**Age at diagnosis**	
≤65	352
≥65	185
**Grade**	
G1	14
G2	230
G3	207
G4	78
NA	8
**Stage**	
Stage I	269
Stage II	57
Stage III	125
Stage IV	83
NA	3
**Tumor**	
T1	275
T2	69
T3	182
T4	11
**Metastasis**	
M0	426
M1	79
NA	32
**Node**	
N0	240
N1	17
NA	280
