# Supplementary material for: A novel necroptosis-related lncRNAs signature for survival prediction in clear cell renal cell carcinoma
Source: Medicine (Baltimore). 2022 Sep 30;101(39):e30621. doi: 10.1097/MD.0000000000030621 (PMC9524942; doi:10.1097/MD.0000000000030621)
Supplement: Supplementary file 2 [file medi-101-e30621-s002.pdf]

Table S2. 68 necroptosis-related genes.

SIRT1
SIRT2
IPMK
FLT3
DDX58
AXL
HAT1
TRIM11
MYC
PLK1
MPG
TNFRSF1B
CASP8
RNF31
TSC1
PANX1
TLR3
DIABLO
EGFR
MYCN
SLC39A7
FAS
SQSTM1
BACH2
ATRX
HDAC9
ZBP1
MAPK8
BNIP3
RIPK1
ALK
BCL2L11
BRAF
HSPA4
TERT
MLKL
BCL2
ITPK1
CYLD
DNMT1
FADD
TNF

KLF9
IDH2
TRAF2
HSP90AA1
RIPK3
FASLG
CD40
SPATA2
ID1
LEF1
GATA3
CDKN2A
STAT3
TNFRSF21
IDH1
CFLAR
SIRT3
MAP3K7
TARDBP
APP
TNFSF10
OTULIN
TNFRSF1A
USP22
STUB1
TICAM1
