# Supplementary material for: A novel necroptosis-related lncRNAs signature for survival prediction in clear cell renal cell carcinoma
Source: Medicine (Baltimore). 2022 Sep 30;101(39):e30621. doi: 10.1097/MD.0000000000030621 (PMC9524942; doi:10.1097/MD.0000000000030621)
Supplement: Supplementary file 3 [file medi-101-e30621-s003.pdf]

Table S3. Twenty-one necroptosis related genes which were differentially expressed in ccRCC.

Genes	logFC	P.Value	regulated
CDKN2A	5.264035	4.8E-142	Up-Regulated
CD40	1.890455	1.62E-63	Up-Regulated
MLKL	1.949649	4.35E-62	Up-Regulated
FASLG	3.494722	2.91E-58	Up-Regulated
ZBP1	2.931892	2.4E-50	Up-Regulated
PLK1	2.34751	3.46E-49	Up-Regulated
TRAF2	1.032717	2.24E-36	Up-Regulated
TNFRSF1B	1.476339	6.29E-34	Up-Regulated
TNFRSF1A	1.062404	8.41E-32	Up-Regulated
BNIP3	1.487064	2.23E-31	Up-Regulated
AXL	1.616156	2.99E-30	Up-Regulated
MYC	1.775465	1.93E-28	Up-Regulated
FAS	1.278001	7.36E-26	Up-Regulated
EGFR	1.325334	1.9E-19	Up-Regulated
ALK	1.738589	3.96E-19	Up-Regulated
TLR3	1.39178	2.06E-17	Up-Regulated
TERT	2.117859	0.00052	Up-Regulated
GATA3	-3.9622	1.29E-80	Down-Regulated
IDH2	-1.06506	3.07E-31	Down-Regulated
MYCN	-1.88156	1.01E-22	Down-Regulated
BACH2	-1.21092	1.06E-17	Down-Regulated

ccRCC = clear cell renal cell carcinoma, FC = fold change
