# Supplementary material for: A novel necroptosis-related lncRNAs signature for survival prediction in clear cell renal cell carcinoma
Source: Medicine (Baltimore). 2022 Sep 30;101(39):e30621. doi: 10.1097/MD.0000000000030621 (PMC9524942; doi:10.1097/MD.0000000000030621)
Supplement: Supplementary file 4 [file medi-101-e30621-s004.pdf]

TableS4: 140 necroptosis-related lncRNAs.

GENE1	GENE2	P	R
HCP5	FASLG	3.48E-40	0.540549
AL096799.1	TLR3	1.21E-34	0.506037
PSMB8-AS1	MLKL	1.05E-42	0.55501
PSMB8-AS1	TRAF2	9.31E-34	0.500123
PSMB8-AS1	FASLG	9.68E-49	0.587031
LINC01428	TLR3	2.88E-37	0.52287
SHANK3	MYCN	1.47E-41	0.54852
SHANK3	MLKL	3.79E-50	0.594014
MIR155HG	ZBP1	7.25E-40	0.538664
MIR155HG	FASLG	6.11E-54	0.612016
AL139287.1	MLKL	1.49E-40	0.542707
WDFY3-AS2	TLR3	1.21E-55	0.619746
WDFY3-AS2	BNIP3	2.14E-36	0.517382
MSC-AS1	BNIP3	1.22E-40	0.543213
AC124854.1	TLR3	2.05E-35	0.511076
HNRNPU-AS1	MLKL	1.21E-39	0.537347
AC138207.5	FASLG	7.29E-34	0.500837
AL132989.1	MLKL	1.74E-35	0.51154
ZNF710-AS1	IDH2	8.03E-94	0.75057
SNHG20	MLKL	1.11E-49	0.591709
AC005674.2	MLKL	2.53E-34	0.503903
AL133371.2	TNFRSF1B	2.04E-49	0.590411
LINC00342	MLKL	5.11E-35	0.508492
AL355803.1	MLKL	1.03E-45	0.571426
AL512791.1	MLKL	2.71E-40	0.541184
USP30-AS1	FASLG	1.05E-81	0.716209
AC007566.1	MLKL	4.86E-38	0.527652
PCED1B-AS1	TNFRSF1B	5.05E-64	0.654863
PCED1B-AS1	ZBP1	1.69E-83	0.721641
PCED1B-AS1	FASLG	1.22E-93	0.750089
SH3BP5-AS1	MLKL	3.57E-37	0.522282
AC011472.1	MLKL	8.81E-34	0.500283
AC018755.4	TNFRSF1B	2.34E-36	0.517136
AC079015.1	FASLG	1.70E-48	0.585804
ARHGAP27P1-BPTFP1-KPNA2P3	MLKL	3.08E-46	0.57418
ITGB2-AS1	ZBP1	2.65E-41	0.547061
ITGB2-AS1	FASLG	3.59E-35	0.509491
AC005104.1	MLKL	4.40E-39	0.533992
AC025171.2	MLKL	7.11E-34	0.50091
AC005261.4	CDKN2A	6.64E-34	0.501109

AL365361.1	ZBP1	3.35E-53	0.608599
AL596325.2	GATA3	3.31E-36	0.51617
AC108134.3	MLKL	4.13E-47	0.578728
AC099850.4	AXL	1.60E-58	0.632331
AC099850.4	PLK1	5.55E-88	0.734678
AC093110.1	MLKL	8.59E-41	0.544101
AL021707.3	MLKL	2.81E-40	0.54109
AL157394.1	FAS	1.80E-43	0.559284
ZNF32-AS2	MLKL	5.23E-40	0.539506
RUSC1-AS1	MLKL	7.16E-36	0.514027
AC116366.2	MLKL	8.98E-34	0.500229
AL031670.1	MLKL	3.94E-34	0.502626
AC138028.4	MLKL	1.20E-47	0.581484
AL121944.2	GATA3	2.45E-53	0.609229
AC109460.2	MLKL	3.47E-40	0.540555
AC015911.3	ZBP1	2.72E-35	0.510277
AC015911.3	FASLG	5.54E-50	0.593205
AC008105.3	ZBP1	1.05E-50	0.596726
WAKMAR2	MLKL	2.23E-48	0.58521
AC243960.1	ZBP1	4.13E-92	0.746032
AC243960.1	FASLG	1.15E-71	0.683257
AC002091.2	TNFRSF1B	6.07E-41	0.544977
AC130469.1	GATA3	2.12E-56	0.62311
AC004921.1	FASLG	6.61E-35	0.507757
EGFR-AS1	EGFR	2.69E-42	0.552721
AC005253.1	MLKL	8.07E-37	0.520061
AL022328.1	MLKL	1.03E-34	0.506498
LINC01150	TNFRSF1B	5.69E-36	0.514668
AC006369.1	FASLG	7.52E-44	0.56137
AC004585.1	ZBP1	2.76E-58	0.631311
AC004585.1	FASLG	8.38E-112	0.792916
AC027601.3	MLKL	8.22E-41	0.544212
TRG-AS1	TNFRSF1B	4.16E-42	0.551651
TRG-AS1	ZBP1	4.80E-51	0.59838
TRG-AS1	FASLG	6.15E-120	0.80931
LINC00861	ZBP1	1.55E-50	0.59591
LINC00861	FASLG	1.22E-44	0.565672
AC013553.3	GATA3	1.67E-57	0.627941
AC116667.1	MLKL	8.41E-35	0.507071
AF127936.5	FASLG	3.09E-35	0.509916
AC009509.5	GATA3	4.06E-36	0.515605
AC004687.1	ZBP1	5.73E-37	0.520996
AL683807.1	ZBP1	1.20E-38	0.531364

AL021707.7	GATA3	3.93E-45	0.568316
AD001527.1	GATA3	3.83E-60	0.639168
AC002091.1	TNFRSF1B	1.68E-55	0.6191
IL10RB-DT	TNFRSF1B	9.29E-36	0.513299
LINC02084	ZBP1	5.06E-57	0.625843
LINC02084	FASLG	1.36E-99	0.76518
AC093001.1	GATA3	4.11E-84	0.723474
AC105020.5	GATA3	3.73E-50	0.594044
AC067817.2	GATA3	3.14E-66	0.663384
AC012186.2	GATA3	3.36E-46	0.57398
AL391845.2	MYCN	2.04E-35	0.511091
AL391845.2	MLKL	7.08E-35	0.507559
AC096733.3	GATA3	2.05E-57	0.627557
AC015819.1	TNFRSF1B	8.66E-49	0.587275
AC145098.1	TNFRSF1B	2.18E-61	0.644305
AL157935.3	ZBP1	7.92E-36	0.513744
AL023653.1	ZBP1	3.44E-53	0.608544
AL023653.1	FASLG	1.09E-41	0.549262
KIF1C-AS1	ZBP1	5.33E-36	0.514851
AL731571.1	MLKL	2.37E-43	0.558626
AC011899.2	TNFRSF1B	5.62E-64	0.654678
AC011899.2	FASLG	2.76E-35	0.510236
AC005740.4	MLKL	5.05E-47	0.578273
CASC2	TLR3	1.35E-45	0.5708
CASC2	FAS	2.45E-52	0.60455
AC114939.1	GATA3	4.24E-65	0.659053
CEP250-AS1	ZBP1	1.49E-48	0.586084
AL023881.1	GATA3	4.81E-39	0.533758
AL031846.2	MLKL	9.67E-38	0.525808
LINC02585	GATA3	1.32E-65	0.661001
LINC00921	MLKL	9.93E-50	0.591954
AC010519.1	GATA3	1.61E-47	0.580833
LINC01772	MLKL	1.20E-42	0.554692
AF111169.3	MLKL	4.00E-42	0.551745
AC009090.1	GATA3	2.56E-76	0.699095
ATP2B1-AS1	GATA3	6.61E-39	0.532926
LINC00426	TNFRSF1B	1.14E-34	0.506208
LINC00426	ZBP1	4.81E-73	0.688056
LINC00426	FASLG	3.96E-112	0.793601
AL590764.1	TNFRSF1B	4.80E-58	0.63028
AL590764.1	ZBP1	5.99E-46	0.572658
AL590764.1	FASLG	2.63E-72	0.685503
AC007743.1	MLKL	1.49E-34	0.505425

AC002550.2	GATA3	3.67E-55	0.617578
LINC00926	ZBP1	3.46E-35	0.509594
LINC00528	ZBP1	1.86E-52	0.605112
LINC00528	MLKL	3.66E-36	0.51589
AC012645.3	ZBP1	3.33E-70	0.678065
AC012645.3	FASLG	2.71E-52	0.604341
AC010618.2	GATA3	2.60E-46	0.574566
AL135818.1	ZBP1	2.77E-45	0.569131
AL135818.1	FASLG	1.59E-116	0.802618
AC087301.1	GATA3	2.46E-68	0.671267
AC010463.2	GATA3	1.31E-59	0.636928
AC004865.2	TNFRSF1B	1.51E-39	0.53678
AC004865.2	ZBP1	3.99E-65	0.659153
AC004865.2	FASLG	5.36E-74	0.691321
NARF-IT1	MLKL	5.77E-35	0.508143
VPS9D1-AS1	PLK1	6.12E-34	0.501344
AC079907.1	GATA3	5.76E-44	0.562003
RP11-680G24.5	GATA3	1.37E-45	0.570761
AC008764.6	GATA3	2.91E-40	0.541008
AC079313.1	GATA3	1.30E-34	0.505832
SUCLA2-AS1	GATA3	6.26E-47	0.577793
SLC25A5-AS1	IDH2	3.63E-34	0.502864
AC079322.1	GATA3	2.16E-48	0.585273
LINC02773	TNFRSF1B	4.31E-35	0.508972
AC090152.1	FASLG	3.43E-35	0.50962
AC018521.5	GATA3	2.24E-42	0.553175
AL139289.1	GATA3	5.52E-50	0.593212
AP003555.1	GATA3	5.81E-66	0.66237
AL132639.2	GATA3	5.88E-43	0.556427
AC004923.4	GATA3	1.33E-59	0.636908
AL136084.3	MYCN	9.34E-46	0.57164
RP11-524D16__A.3	AXL	5.38E-41	0.54528
RP11-524D16__A.3	PLK1	1.51E-39	0.536777
AL357992.1	PLK1	2.21E-37	0.523581
AC005911.1	GATA3	3.16E-64	0.65566
AP003170.3	GATA3	7.58E-54	0.611588
AC112491.1	GATA3	1.86E-45	0.570047
LINCMD1	TLR3	4.45E-34	0.502272
AC008731.1	GATA3	8.17E-40	0.538359
AC020913.3	GATA3	2.72E-58	0.631342
AP000695.1	AXL	8.75E-40	0.538183
AL035446.1	PLK1	4.33E-34	0.502352
AC124017.1	GATA3	4.98E-53	0.607795

AC107057.1	GATA3	1.77E-42	0.553747

lncRNAs = Long noncoding RNAs
