# Supplementary material for: A novel necroptosis-related lncRNAs signature for survival prediction in clear cell renal cell carcinoma
Source: Medicine (Baltimore). 2022 Sep 30;101(39):e30621. doi: 10.1097/MD.0000000000030621 (PMC9524942; doi:10.1097/MD.0000000000030621)
Supplement: Supplementary file 5 [file medi-101-e30621-s005.pdf]

Table S5: 53 significant necroptosis-related lncRNAs after univariate Cox analysis

lncRNA	HR	HR.95L	HR.95H	pvalue
AL096799.1	0.885858	0.808799	0.970257	0.009048
LINC01428	0.840515	0.75124	0.940399	0.002425
SHANK3	0.812327	0.713178	0.92526	0.001751
MIR155HG	1.257297	1.111816	1.421813	0.000263
WDFY3-AS2	0.627749	0.525473	0.749932	2.88E-07
AC124854.1	0.751876	0.691745	0.817234	2.00E-11
HNRNPU-AS1	1.27963	1.094208	1.496472	0.002021
LINC00342	1.334087	1.154479	1.541636	9.34E-05
AL355803.1	0.799595	0.690245	0.926269	0.002875
PCED1B-AS1	1.267927	1.060088	1.516514	0.009356
ARHGAP27P1-BPTFP1-KPNA2P3	1.273372	1.08015	1.501159	0.004
ITGB2-AS1	1.310717	1.14023	1.506695	0.000141
AC005104.1	1.328656	1.133607	1.557267	0.000451
AC005261.4	2.165625	1.674304	2.801124	3.96E-09
AL157394.1	1.581246	1.210086	2.066249	0.000788
ZNF32-AS2	1.316016	1.077988	1.606603	0.006982
RUSC1-AS1	1.331378	1.118556	1.584691	0.001279
AL031670.1	1.406279	1.158411	1.707185	0.000568
AL121944.2	0.462939	0.339635	0.631009	1.10E-06
AC008105.3	1.452875	1.246217	1.693803	1.83E-06
AC243960.1	1.358851	1.117893	1.651746	0.002077
AC130469.1	1.311618	1.14191	1.506549	0.000125
AC005253.1	1.296042	1.06463	1.577753	0.009765
AC027601.3	0.627047	0.507861	0.774205	1.43E-05
AC004687.1	1.23065	1.060471	1.428138	0.006273
AL683807.1	1.399954	1.180538	1.66015	0.00011
AD001527.1	1.267463	1.086038	1.479197	0.002637
AC093001.1	1.171615	1.068022	1.285255	0.000799
AC012186.2	1.388287	1.148811	1.677681	0.000684
AL157935.3	1.93581	1.522628	2.461112	6.96E-08
AL023653.1	1.690387	1.314317	2.174062	4.34E-05
KIF1C-AS1	1.858902	1.471975	2.347537	1.92E-07
CEP250-AS1	1.637221	1.206251	2.222167	0.001561
AC007743.1	0.620388	0.511769	0.752061	1.16E-06
LINC00926	1.640356	1.316087	2.044521	1.06E-05
AC010618.2	1.383057	1.157943	1.651935	0.000346
AC004865.2	1.523307	1.159558	2.001163	0.0025
NARF-IT1	1.521508	1.226117	1.888064	0.000138

VPS9D1-AS1	1.633478	1.369735	1.948005	4.72E-08
AC079907.1	1.413801	1.149529	1.738829	0.001039
RP11-680G24.5	1.448553	1.204128	1.742594	8.50E-05
AC008764.6	1.550682	1.280616	1.877701	7.01E-06
AC079313.1	1.308937	1.09419	1.565831	0.003235
AC079322.1	1.329333	1.087178	1.625424	0.005526
AC018521.5	0.550054	0.37591	0.804872	0.002087
AL132639.2	0.530397	0.341973	0.82264	0.004628
AC004923.4	1.320396	1.113709	1.565441	0.001375
AL136084.3	0.757496	0.646723	0.887244	0.000575
RP11-524D16__A.3	1.279807	1.144029	1.4317	1.62E-05
AL357992.1	1.651086	1.424687	1.913464	2.67E-11
LINCMD1	0.733104	0.603192	0.890996	0.00181
AP000695.1	1.230905	1.054471	1.43686	0.008491
AL035446.1	1.239618	1.056096	1.455031	0.008599

lncRNAs = Long noncoding RNAs
